# A cross-sectional analysis of symptom severity in adults with influenza and other acute respiratory illness in the outpatient setting

**DOI:** 10.1186/1471-2334-14-231

**Published:** 2014-05-01

**Authors:** Jeffrey J VanWormer, Maria E Sundaram, Jennifer K Meece, Edward A Belongia

**Affiliations:** 1 Center for Clinical Epidemiology and Population Health, Marshfield Clinic Research Foundation, 1000 North Oak Ave, 54449 Marshfield, WI, USA

**Keywords:** Acute respiratory illness, Symptom severity, Influenza

## Abstract

**Background:**

Acute respiratory infections (ARIs) are common in outpatient practice, and the severity of symptoms contributes to the overall burden of illness. We examined the association between a subjective symptom severity score, demographic and clinical characteristics, and presence of laboratory-confirmed influenza among central Wisconsin adults who sought care for ARI during four influenza seasons. We hypothesized that adults with laboratory-confirmed influenza would rate their symptoms as more severe relative to adults without influenza, and vaccinated adults with influenza would rate symptoms as less severe than those who were not vaccinated.

**Methods:**

Patients with acute respiratory illness, including feverishness or cough symptoms ≤ 7 days duration, were prospectively enrolled and tested for influenza by reverse transcription polymerase chain reaction (RT-PCR) during influenza seasons 2007–08 through 2010–11. Perceived severity was self-rated during the enrollment interview for eight symptoms, on a scale of 0 (absent) to 3 (severe). Scores for each symptom were summed to generate a combined severity score ranging from 1 to 24 for each individual. The association between influenza test result and severity score was examined using linear regression.

**Results:**

There were 2,374 individuals included in the analysis, including 324 with RT-PCR confirmed influenza. The mean symptom severity score was 12.3 (±4.1) points, and the most common symptoms were cough (92%), fatigue (91%), and nasal congestion (84%). In the final adjusted model, influenza infection was the strongest independent predictor of higher severity score, with a mean increase of 1.7 points compared to those who were influenza negative (p < 0.001). Among adults with influenza, the association between influenza vaccination and symptom severity was modified by age (p < 0.001). In adults ≥ 65 years old with RT-PCR confirmed influenza, symptom severity was 31% lower in those who were vaccinated as compared to those who were not vaccinated.

**Conclusions:**

Influenza is associated with more severe symptoms of acute respiratory illness. The association between influenza vaccination and reduced symptom severity in older adults should be confirmed and explored further in other populations and seasons.

## Background

Acute respiratory infections (ARIs) collectively represent the most common seasonal illness, affecting adults three times annually on average [1,2]. Most ARIs are caused by viruses, including influenza [2]. Studies addressing the burden of illness from viral ARI have historically focused on high-risk populations [3-7] and severe outcomes such as emergency room visits [8], hospitalizations [9-12], and death [13,14]. However, the severity of ARI symptoms in individuals seeking and receiving outpatient care also contributes to the overall illness burden [15]. Such individuals may not experience severe health complications, but their illnesses nevertheless account for a significant economic burden in lost workplace productivity [16]. Because symptom severity is often a determining factor in seeking healthcare for ARI, it underlies billions of dollars in direct medical care expenditures in the United States [17]. Many large observational studies on ARI and influenza-like illness focus on individuals seeking ambulatory medical care, thus a better understanding of the severity of ARI symptoms may improve the interpretability of results of such studies.

From both an economic and public health perspective, influenza is one of the most important viruses because it may cause more severe symptoms than other respiratory viruses. The severity of seasonal influenza symptoms compared to non-influenza ARI has not been extensively studied though. One study reported an association between increased symptom severity in patients with laboratory confirmed 2009 pandemic influenza compared to seasonal influenza [18]; limited evidence also suggests that influenza ARI may be more severe than ARI due to other causes [19]. In an influenza challenge study, symptom severity was associated with increased magnitude of influenza virus shedding [20]. Given its role in generating an immune response and the production of antibodies, influenza vaccination may mitigate the severity of some influenza symptoms. Evidence of this hypothesized impact is incomplete, however, with one study of hospitalized influenza cases demonstrating fewer deaths and ICU admissions (used as markers of severe clinical complications) among those who received a seasonal influenza vaccination [21].

It is well established that ARIs, including influenza, do not affect all patients uniformly [22], yet little is known about host factors and virulence characteristics that are associated with specific ARI symptoms. We had the opportunity to examine the severity of symptoms, along with demographic and clinical features, in adults presenting to an outpatient facility with ARI. Because influenza may present with more severe features relative to ARI of other etiologies, we hypothesized that symptom severity would be rated higher among adults with RT-PCR confirmed influenza. A secondary hypothesis was that influenza vaccination was associated with reduced symptom severity among participants with confirmed influenza.

## Methods

### Design and sample

A cross-sectional analysis was employed using data from annual studies of influenza vaccine effectiveness (VE) among community-dwelling residents of a 14 zip code area surrounding Marshfield, Wisconsin. The VE study methods are described in more detail in previous publications [23]. Briefly, patients in this population were screened and enrolled by trained research coordinators during or directly after an encounter for acute respiratory illness with symptoms of fever or cough. During the 2007–08 season, patients reporting chills were also eligible for enrollment. Potential participants with illness duration >7 days were excluded to minimize false negative influenza test results. Research coordinators used an electronic appointment system to screen for chief complaints and identify potential participants in primary care departments at the Marshfield Clinic main campus. Eligible patients were also recruited from the Emergency Department and an adjacent hospital; individuals enrolled at hospital admission were excluded from this analysis. Patients who could not be approached during their clinical encounter were contacted by phone on the following day if they received an International Classification of Disease (Version 9) (ICD-9) diagnosis code indicating acute respiratory illness. Those who met eligibility criteria were invited to participate.

Each adult participant was interviewed at the time of enrollment to determine illness onset date, and the presence and severity of symptoms. Nasopharyngeal swabs or combined nasal and oropharyngeal swabs were also obtained as part of the enrollment interview and tested for influenza by reverse transcription polymerase chain reaction (RT-PCR). Nucleic acid was extracted from samples using the Roche MagNA Pure Total Nucleic Acid Kit (Roche Diagnostics, Indianapolis, Indiana), and RT-PCR was performed using the LightCycler® Real-Time PCR System (Roche Diagnostics, Basel, Switzerland). The U.S. Centers for Disease Control and Prevention Influenza Division provided sequence information for RT-PCR primers and probes. The TaqMan®-based RT-PCR assay detects two highly-conserved influenza genes: the matrix gene of influenza A and the non-structural gene of influenza B. A human RNase P gene served as a positive control for human nucleic acid. For a subset of study participants with RT-PCR confirmed influenza, cycle threshold (Ct) values were analyzed. Ct values describe the number of times that DNA must be replicated in a particular sample in order to reach a level detectable by a PCR system. Ct values have therefore been used as a surrogate marker of viral titers. For a given respiratory sample, a lower Ct value indicates a greater amount of viral material present [24,25].

The recruitment period generally corresponded to periods of influenza transmission from 2007–08 to 2010–11. The length of enrollment ranged from 10 weeks in 2007–08 to 26 weeks in 2009–10 (December to May). For this analysis, we excluded enrollments occurring during the 2009 pandemic wave (October-November), since the clinical features and symptom severity scores have been previously reported [18]. The VE study procedures were approved by the Marshfield Clinic Institutional Review Board and informed consent was obtained from all participants.

### Symptom severity score

The primary outcome for this analysis was ARI symptom severity score, which was assessed at (and reflected symptom severity at) the time of the enrollment interview and before any RT-PCR results were disclosed. Symptom severity score was assessed using a subjective self-rating of eight symptoms, including two upper respiratory (nasal congestion, sore throat), two lower respiratory (cough, wheezing), and four systemic symptoms (feverishness, fatigue, headache, muscle aches). Participants rated each of their symptoms on an ordinal scale that included response options of absent (0), mild (1), moderate (2), or severe (3). These symptom ratings were summed to create a symptom severity score with a possible range of 1–24 points, where higher scores indicated greater perceived severity of symptoms. All participants had a minimum score of 1 because either feverishness or cough was required for enrollment in all seasons.

### Predictor variables

The primary predictor variables were influenza RT-PCR result and age. Based on previously observed associations with RT-PCR confirmed influenza in some seasons, other considered exposures included sex, type of health insurance, enrollment season, days between symptom onset and enrollment, number of outpatient visits in the past five years, same-season influenza vaccination status, blood pressure (high [≥140/90 mmHg], borderline [120-139/80-89 mmHg], normal [<120/80 mmHg]), total cholesterol (high [≥240 mg/dL], borderline [200–239 mg/dL], normal [<200 mg/dL]), body mass index (BMI) (obese [≥30 kg/m^2^], overweight [25–29 kg/m^2^], normal [<25 kg/m^2^]), smoking history (current, former, never), and personal history of chronic pulmonary disease, diabetes, and/or cardiovascular disease (CVD). Chronic disease status was determined by the presence of specific ICD-9 diagnostic codes in the electronic health record (list of codes available on request). For BMI, blood pressure, and cholesterol, the most recent recorded value within five years prior to VE study enrollment date was used. BMI was calculated as weight in kilograms divided by height in meters squared. Influenza vaccination status (including vaccination date) was ascertained from an online immunization registry that is linked to clinical and non-clinical vaccination records from the region. This registry has been demonstrated to capture over 95% of influenza vaccines administered in this study population [26].

### Analyses

Linear regression was performed to examine the associations between symptom severity score, influenza, age, and other covariates. Data from all seasons were combined and symptom severity score was modeled as a continuous variable since it was normally distributed. Collinearity between exposure variables was assessed by examining the variance inflation factors and condition index statistics [27]. No collinearity issues were discovered; therefore univariate models were first created to assess the crude association between each exposure and symptom severity score. A fully adjusted multivariable model was then fit with all exposures and all possible two- and three-way interaction terms between influenza, age, sex, and vaccination status. Along with influenza and age, exposures with a significant (p < 0.05) association in the univariate models were considered for inclusion in the final, reduced multivariable model. All considered exposure terms were initially entered simultaneously and manual, backward selection criteria were applied to sequentially eliminate exposures that were not independently associated with symptom severity score. Secondary analyses of Ct values in participants with RT-PCR confirmed influenza included non-parametric t-tests to examine Ct differences by vaccination status. Analytical procedures were conducted using SAS Version 9.3 (Cary, NC).

## Results

**Figure 1:**
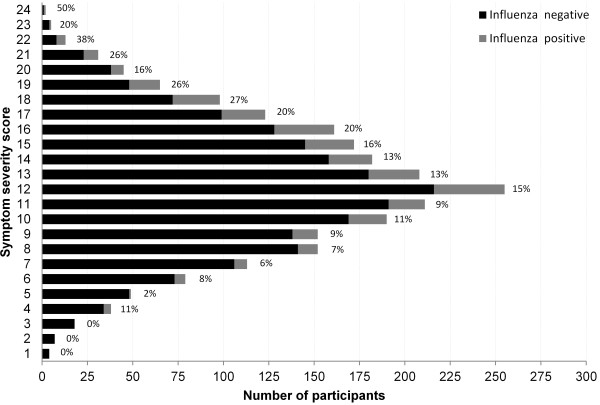
**Number of participants who were influenza positive and negative by symptom severity score.** The percentages above each bar correspond to the percent of participants who were influenza positive at each symptom severity score value.

There were 2,374 participants who met all eligibility criteria and were included in the analysis. Two-thirds of participants were female, and the majority was under age 50 years (55%), with 29% and 16% in the 50–64 years and ≥65 years groups, respectively (Table 1). Mean (±SD) symptom severity score was 12.3 (±4.1) points overall, with significantly decreased symptom severity in older age groups (p < 0.001). Influenza was significantly less common in older age groups (p = 0.012), with rates of RT-PCR confirmed influenza being 15%, 13%, and 9% in participants age 18–49, 50–64, and ≥65 years, respectively. The most common symptoms were cough (93%), fatigue (91%), nasal congestion (84%), headache (76%), sore throat (76%), muscle aches (70%), fever (69%), and wheezing (49%). The median severity rating for each symptom, when present, was ‘moderate.’ In unadjusted univariate models, higher symptom severity score was significantly associated with younger age group, female gender, a greater number of recent ambulatory visits, not being vaccinated for influenza, borderline high cholesterol, higher BMI, and current smoking (see Table 1). Influenza varied significantly by enrollment season and time interval between symptom onset and enrollment. Compared to those with symptom severity scores below the median, more participants tested positive for influenza among those with symptom severity scores above the median (Figure 1).

**Table 1:** Descriptive characteristics of study participants with acute respiratory illness during four influenza seasons

**Characteristic**	**N (%)**	**Mean ± SD symptom severity score**	**P-value**^**1**^
Age (years)			
18-49	1299 (54.7)	12.7 ± 4.1	< 0.0001
50-64	676 (28.5)	12.3 ± 4.0	
≥ 65	399 (16.8)	10.8 ± 4.2	
Gender (female)	1591 (67.0)	12.6 ± 4.1	< 0.0001
Health insurance			
Private only	1200 (50.6)	12.2 ± 3.8	0.56
Public-assisted	1082 (45.6)	12.4 ± 4.4	
vNone	92 (3.9)	12.2 ± 4.5	
Study season			
2007-08	249 (10.5)	13.0 ± 4.1	0.006
2008-09	672 (28.3)	12.4 ± 4.4	
2009-10	838 (35.3)	12.0 ± 4.0	
2010-11	615 (25.9)	12.2 ± 4.0	
Days elapsed between symptom onset and date of enrollment			
0	67 (2.8)	10.0 ± 4.1	< 0.0001
1-2	654 (27.6)	12.3 ± 4.0	
3-4	798 (33.6)	12.5 ± 4.1	
5-6	534 (22.5)	12.2 ± 4.1	
≥ 7	321 (13.5)	12.1 ± 4.2	
Number of ambulatory care visits within the past 5 years			
0-20	334 (17.4)	11.6 ± 4.0	0.009
21-40	603 (31.4)	12.0 ± 4.0	
41-60	474 (24.7)	12.4 ± 4.1	
61-80	313 (16.3)	12.4 ± 4.3	
81-100	195 (10.2)	12.7 ± 4.1	
≥ 101			
Influenza infection	324 (13.7)	13.9 ± 3.9	< 0.0001
Presence of chronic disease			
Pulmonary disease	296 (12.5)	12.4 ± 4.2	0.43
Diabetes	290 (12.2)	12.1 ± 4.2	0.43
Cardiovascular disease	322 (13.6)	11.8 ± 4.4	0.05
Blood pressure (systolic/diastolic mmHg)			
< 120/80 (healthy)	724 (30.5)	12.3 ± 3.9	0.59
120-139/80–89 (borderline)	1159 (48.8)	12.3 ± 4.2	
≥ 140/90 (high)	491 (20.7)	12.1 ± 4.3	
Total cholesterol (mg/dL)			
< 200 (healthy)	1644 (69.3)	12.1 ± 4.1	0.002
200-239 (borderline)	566 (23.8)	12.7 ± 4.2	
≥ 240 (high)	164 (6.9)	12.7 ± 4.1	
Body mass index (kg/m^2^)			
< 25 (healthy)	465 (19.6)	12.3 ± 3.9	0.01
25-29 (overweight)	648 (27.3)	12.3 ± 4.2	
≥ 30 (obese)	1261 (53.1)	12.1 ± 4.3	
Smoking status			
Never	1259 (53.0)	11.9 ± 3.9	< 0.0001
Former	719 (30.3)	12.5 ± 4.3	
Current	396 (16.7)	12.9 ± 4.4	

^1^P-value corresponds to *t*-test comparing symptom severity score between each characteristic category.

**Figure 2:**
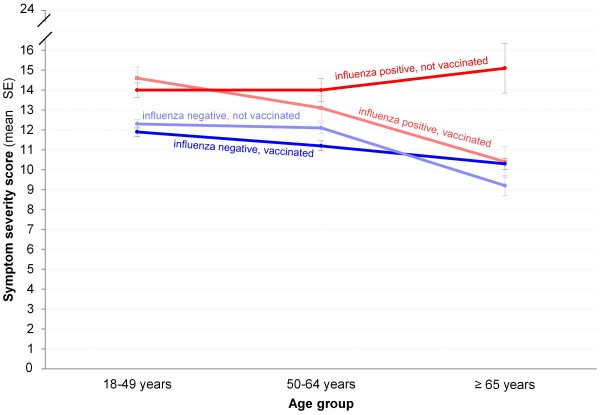
Least-squares adjusted symptom severity scores, stratified by age group, influenza, and vaccination status (N = 2,374).

The main findings from the final adjusted model in Table 2 indicated that female gender, more ambulatory care visits, borderline high cholesterol, obesity, and smoking (both current and former) were associated with significantly higher symptom severity scores. In addition, there was a significant three-way interaction observed between age group, influenza, and vaccination status. Age modified the association between vaccination status and symptom severity in patients who were influenza positive (Figure 2). Mean symptom severity generally declined with increasing age, but remained highest among non-vaccinated individuals with influenza. Among participants ≥65 years old with influenza, mean symptom severity scores were 5–6 points higher in those who were not vaccinated relative to those who were vaccinated or those who did not have influenza (regardless of vaccination status; p <0.001 for all pairwise comparisons). Significant reductions in the severity of cough and feverishness, as well as a decreased presence of wheezing, largely explained the lower symptom severity scores observed in the older, vaccinated group with influenza (Table 3).

**Table 2:** **Multivariable associations between participant demographic and clinical characteristics and symptom severity (N = 2,374)**^**1**^

**Characteristic**	**Difference in average adjusted symptom severity score**^**2**^	**Standard error**	**P-value**
Age (years) (ref.: 18–49)			
50-64	−0.19	0.35	0.51
≥ **65**	**−3.08**	**0.49**	**< 0.0001**
**Female gender**	**0.99**	**0.18**	**< 0.0001**
Days elapsed between symptom onset and enrollment date (ref.: ≥ 7)			
5-6	0.06	0.28	0.82
3-4	0.04	0.26	0.87
1-2	−0.27	0.27	0.31
**0**	**−2.45**	**0.53**	**< 0.0001**
**Number of ambulatory care visits in the past five years**	**0.01**	**0.001**	**< 0.0001**
**Influenza infection**^**3**^	**1.73**	**0.36**	**< 0.0001**
Vaccinated against influenza	−0.39	0.24	0.11
Total cholesterol (mg/dL) (ref.: < 200)			
**200-239**	**0.67**	**0.19**	**0.0006**
≥ 240	0.46	0.32	0.15
Body mass index (kg/m^2^) (ref.: < 25)			
25-29	0.27	0.24	0.27
≥ **30**	**0.61**	**0.22**	**0.005**
Smoking (ref.: Never)			
**Current**	**0.63**	**0.23**	**0.006**
**Former**	**0.73**	**0.19**	**< 0.0001**
Interaction: Age x influenza			
50-64 × influenza infection	0.23	0.72	0.75
≥ **65 × influenza infection**	**4.21**	**1.37**	**0.002**
Interaction: Age × influenza vaccination			
50-64 × vaccinated	−0.48	0.41	0.24
≥ **65 × vaccinated**	**1.45**	**0.57**	**0.01**
Interaction: Influenza infection x influenza vaccination	0.92	0.68	0.17
Interaction: Age x influenza infection x influenza vaccination			
50-64 x influenza infection × vaccinated	−1.02	1.11	0.36
≥ **65 × influenza infection×vaccinated**	**−6.75**	**1.68**	**< 0.0001**

^1^Bolded values denote point estimate was significant (p < 0.05). Significant exposures in the crude models were also considered for inclusion in the final reduced model.

^2^Values are reported as point estimate ± standard error, p-value. Positive values with p < 0.05 indicate a significantly greater symptom severity score relative to the reference category (or 1-unit increase for continuous predictors) and negative values with p < 0.05 indicate a significantly lower symptom severity score relative to the reference category.

^3^Influenza infection refers to infection with either influenza A or B.

**Table 3:** **Presence, absence, and mean severity scores of individual ARI symptoms in study participants with RT-PCR confirmed influenza, stratified by age and influenza vaccination status (n = 324)**^**1**^

	**18-49 years**		**50-64 years**		**≥65 years**		
	**Vaccinated**		**Vaccinated**		**Vaccinated**		
**Symptoms**	**Yes**	**No**	**Yes**	**No**	**Yes**	**No**	***p***
	**(n = 52)**	**(n = 144)**	**(n = 43)**	**(n = 48)**	**(n = 27)**	**(n = 10)**	
Nasal congestion	2.15	1.95	1.74	1.97	1.89	2.13	0.128
Present (%)	90	89	91	81	70	80	0.465
Absent (%)	10	11	9	19	30	20	
Sore throat	2.00	1.97	1.86	1.84	1.67	2.57	0.056
Present (%)	87	76	67	77	56	70	0.112
Absent (%)	13	24	33	23	44	30	
Cough	2.39	2.18	2.30	2.47	2.41	2.80	0.046
Present (%)	98	97	100	98	100	100	0.998
Absent (%)	2	3	0	2	0	0	
Wheezing	1.88	1.57	1.75	1.68	1.85	1.78	0.607
Present (%)	46	53	65	52	48	90	0.045
Absent (%)	54	47	35	48	52	10	
Feverishness	2.17	2.17	1.87	2.11	1.70	2.63	0.016
Present (%)	88	89	91	94	74	80	0.904
Absent (%)	12	11	9	6	26	20	
Headache	2.19	2.11	2.03	1.90	1.58	2.13	0.167
Present (%)	83	82	77	88	70	80	0.504
Absent (%)	17	18	23	13	30	20	
Muscle aches	2.11	2.24	2.15	2.15	1.93	2.43	0.432
Present (%)	87	88	77	96	56	70	0.163
Absent (%)	13	12	23	4	44	30	
Fatigue	2.40	2.34	2.48	2.40	2.25	2.50	0.558
Present (%)	96	94	98	98	89	100	0.955
Absent (%)	4	6	2	2	11	0	

^1^Values for symptom severity scores are reported as mean and p-value, among participants where the symptom was present. Values for the presence/absence of each symptom are reported as percent of column total, and p-value for chi-square test comparing symptom between each age/vaccination group.

In a secondary analysis, Ct values were examined from RT-PCR confirmed influenza positive samples. Due to the relatively small available sample for this analysis, Ct values were dichotomized as having a high or low viral load based on the median value of 25.7. Among all influenza positive participants, the median severity score was 15 for those with a high viral load and 13 for those with a low viral load (p = 0.09). Among adults ≥ 65 years old, the median severity score was 12 for those with a high viral load and 12 for those with a low viral load (p = 0.27). There was no association between vaccination status and viral load among all participants with influenza (p = 0.62). Among vaccinated adults ≥65 years old, the median severity score was 12 and 10, respectively, for those with a high and low viral load (p = 0.32).

## Discussion

Symptom severity can serve as a useful and complementary measure of respiratory disease impact among individuals seeking care for respiratory illness in the outpatient setting. Perceived ARI symptom severity was greatest in younger adults, women, and individuals with RT-PCR confirmed influenza in this study. As expected, influenza infection was most closely associated with increased symptom severity. The presence of diagnosed CVD, pulmonary disease, or diabetes had little association with symptom severity, though some disease risk factors (e.g., obesity, smoking, dyslipidemia) were modestly associated with higher symptom severity scores.

The subgroup analysis of influenza positive participants identified an association between influenza vaccination and reduced symptom severity in older age groups. Compared to ≥ 65 year old adults who were influenza positive but not vaccinated, perceived symptom severity was 31%-39% lower in older adults who were influenza negative or who were vaccinated. This difference was primarily driven by reductions in the severity of cough and feverishness, as well as reduced presence of wheezing, in those who received the vaccine. However, there were only 10 unvaccinated individuals with influenza who were ≥ 65 years old; therefore these findings could reflect random variation as a function of the small sample size. The biological basis for reduced symptom severity in vaccinated older adults (but not in younger adults) is unclear and largely speculative at this point. The humoral and cell-mediated immune response to natural influenza infection and influenza vaccination declines with increased age, thus the potential vaccine benefits in terms of symptom mitigation would seem to be more likely in younger, rather than older, individuals [28,29]. It is also possible that the observed reductions in systemic (fever) and lower respiratory (wheezing) symptoms could have resulted from reduced influenza virus replication in vaccinated versus unvaccinated older adults. However, our analysis of RT-PCR Ct values as a surrogate measure of viral load did not reveal any significant associations with either severity score or vaccination status. There may also be perceptual reasons that explain why those in older age groups or who received a prior influenza vaccination perceived their symptoms differently. Further research is needed to understand the relationship between vaccination, influenza severity, and virus shedding in older adults.

Strengths of this study included recruitment from a defined population cohort over multiple seasons, systematic screening and enrollment, standardized reporting of severity at the time of enrollment, use of a validated immunization registry, and confirmation of influenza using a highly sensitive and specific RT-PCR test. From a limitations perspective, individuals who did not receive medical attention for their ARI were not recruited, thus results are not necessarily generalizable to all influenza illness cases in the community. From a measurement perspective, variations of the ARI symptom severity metric used in this study have been used in numerous other investigations [30-38] and have been previously correlated with influenza positive status [19] and viral shedding [20], but have not been formally validated and remain a subjective measure of symptom perceptions that may be subject to recall and self-presentation biases, as is common with some other self-reported metrics [39]. This is a particular concern in some subgroup comparisons where symptom severity expectations may vary as a result of perceptual priming during study enrollment (e.g., different beliefs on how influenza vaccination should impact symptoms). Additional research is also needed in this area to better understand self-reported ARI symptom severity and its relationship to functional disability and serious medical complications. In addition to influenza, future studies should also examine symptom severity for other specific pathogens and virus infections such as RSV, rhinovirus, and human metapneumovirus.

## Conclusions

Influenza infection was the strongest predictor of perceived symptom severity among individuals with medically attended ARI over multiple seasons. There was also a modest reduction in perceived severity among vaccinated vs. unvaccinated individuals ≥65 years old with confirmed influenza. However, these findings could be influenced by random effects or measurement biases, thus further research to confirm this finding in other populations and seasons is warranted.

## Competing interests

E.A.B., J.K.M., and M.E.S. have grant support from Medimmune, LLC. All other authors: No reported conflicts.

## Authors’ contributions

E.A.B. designed and conducted the original influenza vaccine effectiveness study; J.J.V. designed the research and statistical procedures for this analysis. J.J.V. and M.E.S. conducted statistical analysis. J.J.V. and M.E.S. wrote the manuscript with input from E.A.B. and J.K.M. on statistical analysis methods, tables and figures, and manuscript structure. All authors read and approved the final manuscript.

## Pre-publication history

The pre-publication history for this paper can be accessed here:

http://www.biomedcentral.com/1471-2334/14/231/prepub
